# Formative research in the development of a salutogenic early intervention home visiting program integrated in public child health service in a multiethnic population in Norway

**DOI:** 10.1186/s12913-018-3544-5

**Published:** 2018-09-27

**Authors:** Maria J. Leirbakk, Johan Torper, Eivind Engebretsen, Jorunn Neerland Opsahl, Paula Zeanah, Jeanette H. Magnus

**Affiliations:** 1 Department of Health Sciences, University of Oslo, Harald Schjelderups hus, Forskningsveien 3a, 0373 Oslo, Norway; 2 Agency for Health, City of Oslo, Storgata 51, 0182 Oslo, Norway; 3 Department for Primary Health and Social Services, City of Oslo, City Hall, NO-0037 Oslo, Norway; 4 Department of Health, City of Oslo, District Stovner, 0985 Oslo, Norway; 5 College of Nursing and Allied Health Professions and Cecil J. Picard Center for Child Development and Lifelong Learning, University of Louisiana at Lafayette, 200 East Devalcourt Street, Lafayette, LA 70506 USA; 6 Faculty of Medicine, University of Oslo, Klaus Torgårds vei 3, Sogn Arena, 0372 Oslo, Norway; 7 Department of Global Community Health & Behavioral Sciences, Tulane School of Public Health and Tropical Medicine, 1440 Canal Street, New Orleans, LA 70112 USA

**Keywords:** Early intervention, Formative research, Home visits, Maternal and child health, Program development, Salutogenesis

## Abstract

**Background:**

Few early intervention programs aimed at maternal and child health have been developed to be integrated in the existing Child Health Service in a country where the service is free, voluntary and used by the majority of the eligible population. This study presents the process and the critical steps in developing the “New Mothers” program.

**Methods:**

Formative research uses a mixed method, allowing us to obtain data from multiple sources. A scoping review provided information on early intervention programs and studies, clarifying key elements when framing a new program. Key informant and focus group interviews offered insight of existing challenges, perceptions, identified power structures and offered reflections germane to the identified framework, securing user involvement at all stages. Monthly meetings with the project group enabled feedback loops for the data, securing program advancement.

**Results:**

The “New Mothers” program was formed based on a salutogenic theory, emphasizing resistance and strengths. Public health nurses in the existing Child Health Service were to offer universally all first-time mothers and children home visits from gestational week 28 until the child reached 2 years, with motivational interviewing and empathic communication as methods to mentor the mothers, help them identify their strengths and resources, and provide support and information.

**Conclusions:**

Using formative research as mixed method ensures incorporation of detailed information from multiple resources when an early intervention program is developed. This method secured program appropriateness, both culturally and at system level, when integrating new elements in the existing service.

## Background

Historically, Norway was a homogenous society. However, over the last decade, significant migration has created a complex society with immigrants from over 200 countries [1], and consequential created a society which challenges the health and social services, prompting organizational change and action. New strategic and political guidelines in recent White papers directed at the municipalities responsible for managing the Primary Health Care service (PHC) [2, 3] focused on emphasizing early health promotion and risk prevention, especially related to children and adolescents due to gross social inequality in their health status [4].

The Stovner District, one of Oslo’s 15 districts, experienced particularly alarming statistics in 2016. Stovner, where 55% of the inhabitants were immigrants or had parents of non-western background [5], had the highest child poverty rate in Norway, with almost every third child growing up in a poor household [4], compared to every 10th child in 2000 [6]. Every third student dropped out of high school and one in four above 20 years had only primary school education [4]. In addition there was steep increase in use of secondary services, especially within the Child Welfare Services (CWS) during the last decade. The CWS have a statutory obligation to ensure that children and youth living in conditions that might be detrimental to their health and development receive the necessary assistance and care.

Four years ago, the administrators of the Stovner district invited their leaders in health-related positions and researchers to a series of meetings to provide ideas and advice on how to accommodate both incremental social service demands and the new national policy guidelines. The first meeting called for identifying innovative solutions for the reduction of downstream and long-term expensive secondary measures such as CWS. The desire was to anchor new initiatives in the Child Health Service (CHS). The CHS is established by law as a key organizational component of the PHC in Norway. Its primary goal is to secure an accessible, low threshold, and free health care service for pregnant women, children and adolescents under the age of 20, focused on health promotion and risk prevention. The service is run by specialized Public Health Nurses (PHNs), midwives, and family medicine doctors, and is used nationally by 98% of the eligible population [7]. During pregnancy, midwives offer women nine health examinations from gestational week 8. From birth to age 4 years, children are offered 14 routine health examinations provided by PHNs and family medicine doctors. The service continues for the school children until completion of 12th grade. The main goal is ensuring an optimal development and growth trajectory in the children, as well as providing guidance to the parents [8].

A new program had to be acceptable to the users of the CHS, endorsed by the PHNs, and integrated within the current Norwegian CHS structure, context and system. New political guidelines required user involvement, so the process needed to assure participation of both users of the CHS and the PHNs. Users are often involved in evaluations, but uncommonly in the process of development of new services [9]. The short-term goal of the program was to develop and pilot a home visiting program integrated in the current CHS, and long-term reduction of the use of secondary interventions like CWS. The aim of this study was to develop a program for early intervention to be integrated in the current CHS.

## Methods

**Fig. 1:**
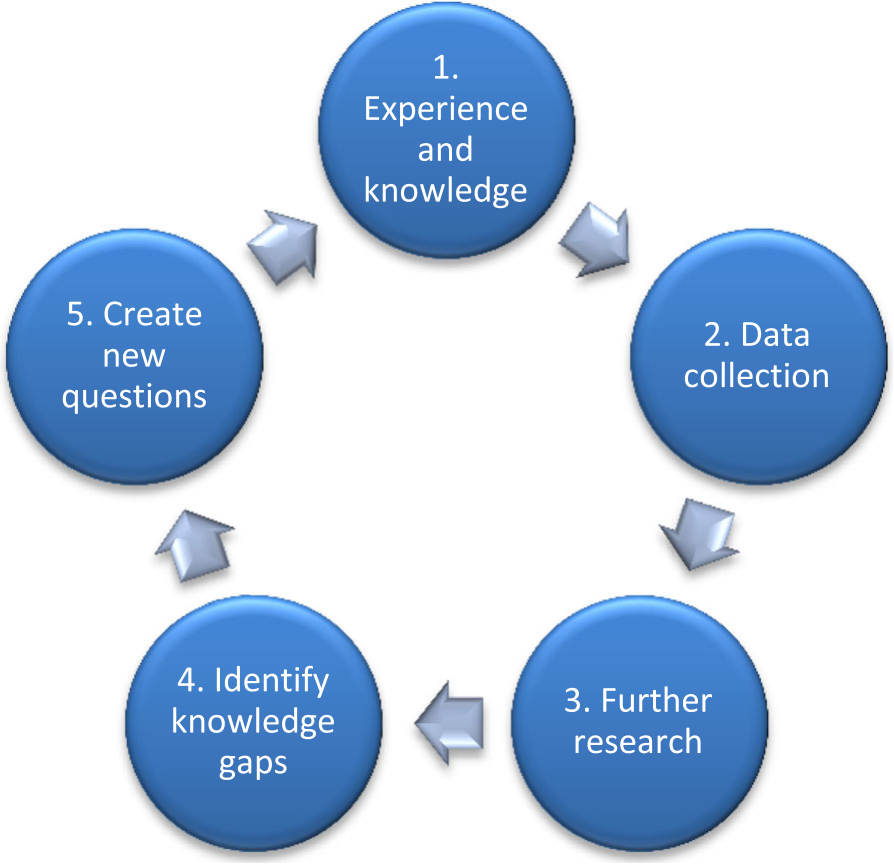
The process cycle of formative research

A feasibility study clarified the acceptance and interest of a home visiting program in the district. Rather than imposing a pre-determined approach, a formative research design was chosen where data obtained from multiple sources allowed analysis, identification and clarification of applicable and critical key elements and perspectives as they emerged. This design also allowed identification of relevant aspects associated with equality and equity within the CHS. The major role of formative research is to assure program appropriateness, both culturally and demographically [10]. It has been applied to a range of clinic-, school-, community- and population-based interventions [11–14], and the value well documented in the literature [10, 15], and emphasized by the Medical Research Council [16]. Based on the iterative formative research process, we developed a cycle, consisting of 5 steps (Fig. 1).

**Fig. 2:**
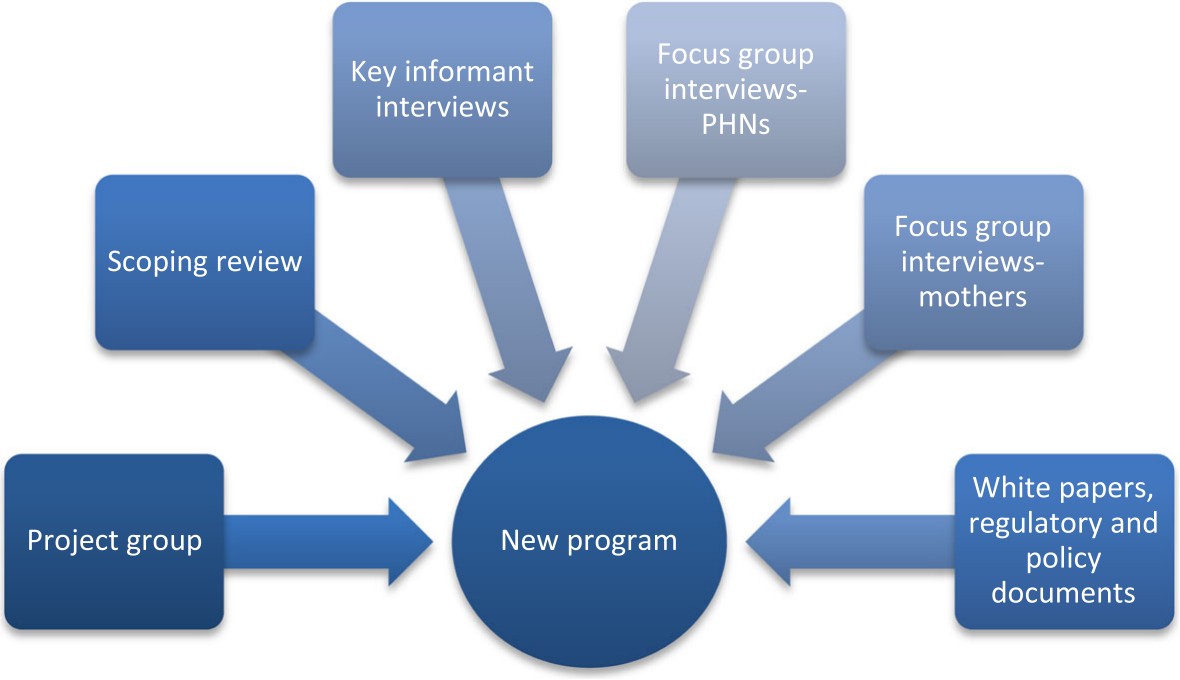
Subject and participants of the study

The process is a spiral continuing to grow, never ending up at its initial base, but constant developing. It is inspired by Gadamer’s theory of a hermeneutic circle, describing the process of understanding and interpreting observations and texts [17]. This theory claims that no observation or description is free from the effects of the observer’s experiences, pre-suppositions, and projections of his or her personal values and expectations [18]. Hence, a formative evaluation must systematize and actively draw on the knowledge and experiences of the different participants (Fig. 2).

### Data collection

#### Scoping review

Scoping reviews of home based early intervention, and the use of participatory research in CHS intervention development, identified existing knowledge, strategies and impact. The reviews identified relevant programs, but with elements not suitable for the Stovner District due to cultural or organizational differences, differences in intended outcome, target groups or framing premises. The reviews focused on existing and evaluated programs, program theory, approach, outcomes and methods. Seeking to answer the who, why and how within the traditions of existing programs, we narrowed the search to interventions carried out during early childhood using home visits directed at advancing maternal and child health. We also searched for additional literature describing development of new services within existing service. In this paper we only present the data the project group identified as relevant for the development of the new program. In addition, a host of documents, including Norwegian white papers, regulatory, and policy documents were reviewed to understand and clarify obligations and regulations affecting the CHS service.

**Fig. 3:**
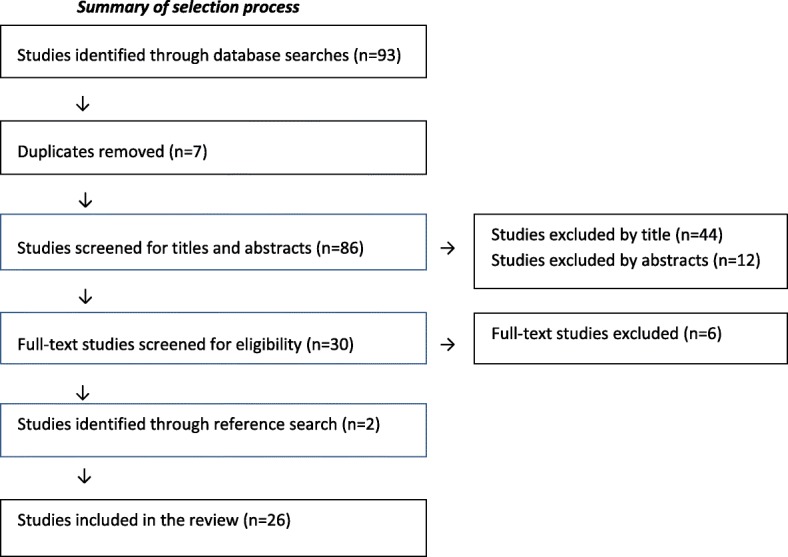
Flow diagram of the number of studies identified, included and excluded by scoping review

Using “Early intervention (mesh term) AND nurs* AND maternal and child (mesh term) AND home visit* (mesh term)”, we carried out a search through the Pubmed, Cochrane, and EMBASE databases. Figure 3 contains the initial search strategy employed. Studies on evidence-based early intervention programs where framework or approach were missing prompted extended searches at program websites. The review explored the contingencies within the studies to address the implementation questions for the new program. We excluded studies with specific focus, such as overweight, specific diseases or children over age two.

The scoping review identified 26 early intervention studies in total, from USA [19–29], Australia [30–36], Canada [37–39], United Kingdom [40, 41], New Zealand [42] Germany [43] and Sweden [44]. The majority of the studies examined evidence-based intervention programs. Others were described as projects [30, 31, 39].

#### Participants

The project group consisted of senior administrative staff from the CHS, the Chief District medical Officer and Assisting District Director, a professor and an advisor from the University of Oslo, and the Program Coordinator. Three PHNs from the district CHS were actively involved through regular meetings and worked closely with the project group. In addition, 18 randomly chosen mothers who used the districts CHS participated in focus group interviews.

#### Process meetings

The project group had monthly meetings, which clarified members’ knowledge, experience, viewpoints, and identified knowledge gaps using the process steps (Fig. 1). Assumptions and earlier conclusions were revised as new questions emerged. Meetings ended by delegating task responsibilities and identifying the next steps of the program development, documented through process minutes. The description of the preliminary program continued to evolve using the formative research cycle.

### User participation

#### Qualitative data collection

As an essential part of formative research, focus groups and key informant interviews assure user involvement at all stages of the process and assist in defining attributes and context of the target groups of interest in the particular setting [13, 45]. This qualitative process supports the hermeneutic cycle, enabling creation of understanding and insight into different meanings and their interpretation [46]. In the current study, both key informant interviews and focus groups were conducted.

Key informant interviews were conducted with members of the project group, and focus group interviews with the three PHNs. The interviews lasted for an hour and clarified cultural and social norms, experiences and identified contingencies valuable for program development [47]. Five focus groups with duration of 1.5 h, with a total of 18 mothers who used the district CHS were held. Group opinions and community norms were explored, experience with the CHS, and perspectives related to parenting and care of infants. This provided valuable insight on behaviors and practices. All interviews and focus groups were led by a moderator and an observer and took place at the district CHS.

### Data analysis

Interviews and focus groups were audio-recorded, transcribed, and analyzed. Together with minutes and protocols, were all available texts analyzed using Systematic text condensation, a descriptive and explorative analysis strategy [48], following four steps: 1) total impression - from chaos to themes; 2) identifying and sorting meaning units - from themes to codes; 3) condensation - from code to meaning; 4) synthesizing - from condensation to descriptions and concepts (Malterud, 2012). Nvivo 10 was used as qualitative data analysis software [49].

## Results

### Premises for a new program

The project group wanted the intervention to be universal to avoid stigmatization and to capture the needs of the mothers individually without an initial high-risk identification. In addition the intervention was to be implemented in the existing CHS. The objective was to offer all first-time mothers additional services by PHNs and establish a trusting and supporting relationship. It was presumed that if the mothers received additional help and support when they had their first child, good strategies, knowledge and habits potentially would persist when they had their next child. The suggestion was to use home visits as the delivery method. It was hypothesized that the balance of power in the relationship between the mother and the PHN would shift positively, when the PHN was a guest in the mothers’ home. In the Norwegian statutory program, the PHNs visit parents once within the first 2 weeks after birth, so they were familiar with conducting home visits. The first key component discussed was which program theory would capture the intention of the Norwegian CHS best, upholding a universal approach. The project group wanted to focus the attention during the home visits on strengths and opportunities within the family and its environment. And hence, a theory had to support such a strategy.

### The program theory

The scoping review identified important key elements of early intervention programs, and highlighted the need of a theoretical foundation. The project group thought that the theory reflecting the desired focus for the new program model was salutogenesis, a theory by Antonovsky claiming that the way people viewed their life influenced their health [50]. People who viewed their life positively managed stressful situations better and were thus able to improve their health. This term he called ‘Sence of Coherence’ (SOC). Through SOC, Antonovsky reasoned it was not the personal resources themselves that were important, but rather the ability to use them (ibid). These resources are described as General Resistance Resources (GRR), and are the cornerstones in the development of a strong SOC. They could be: genetic and constitutional, psychosocial, cultural and spiritual, material, and all be risk preventive and health promotive [51]. In line with the Norwegian universal approach at the CHS, all families are offered the same service, regardless of their risk factors. The universal approach would be supported by incorporating a salutogenic theory, emphasizing resistance and strengths, not potential deficits.

### Public health nurses

Next was to determine if the theory was in accordance with how the PHNs regarded their work. Salutogenesis was not new to the PHNs; one described how their practice reflected a salutogenic theory:

*“Sometimes we have to wrap it up and rather say: “let it go”. Then we have to think: okay, let’s focus at the things that the mother herself thinks she can do.” (PHN).*

The PHNs acknowledged the importance of supporting the mothers. They described how manageability was important to the parents they met, and they (the PHNs) tried to detect, support and focus on parental strengths.

*“The individual, that’s what’s important. That’s the real dilemma at the CHS. We sit there knowing what’s general, we have to adapt to the individual.” (PHN).*

A negative power structure can be created if the CHS treat all users the same, without adapting to the individual. By using home visits as intervention method, the PHNs would meet the families at home, providing a different perspective on family strengths and resistance, through the individual setting reflected in their home.

Mothers’ perspective

A mother in the focus group told how she liked to figure it out by her own describing a more heuristic method, strengthening her self-confidence:

*“You are here to learn from your own mistakes...well this is what I know of myself. I know that I increase my trust in myself when I manage to… yeah, learn by myself” (Focus group mother).*

The mothers shared their experiences on how PHNs at times overwhelmed them with advice on how to improve their parenting skill. At times this felt intrusive, but their respect of the PHNs authority and power prohibited any response. Such an approach would contradict the universality and intended low threshold service at the Norwegian CHS, and the salutogentic theory stressing the importance of how people view their life affects their health.

The PHNs regarded themselves as professionals who could provide guidance and support; the mothers wanted to be able to set the agenda but at the same time have the opportunity to get help when needed. This was in accordance with the salutogenic theory. In addition, the PHNs had experienced how seemingly well-functioning parents also at times needed additional support, in contrast to a program only aiming at high-risk families. A program based on high-risk identification would therefore be a pathogenic program. As “New Mothers” was to be universal, the salutogenic theory would better describe the intention, focus and vision of the intervention program.

### Intervention approach or model

Some of the scoping review studies described their intervention approaches or intervention models as: “Plan to do act” [44], “Stages of change model” [37], “Social learning approach” [42], “Learning to communicate” [32], “Cycle of learning” [36, 44] “Parent adviser”, “Motivational interviewing” [36, 44] and “Strength based approach” [28, 32, 36]. Other studies described their treatment approaches [32, 33, 44]. As a method to captivate and connect with the mothers, the project group decided that the PHNs should anchor the intervention in methods and techniques like Motivational Interviewing (MI) [44] and Empathic Communication [52], as these were familiar to the Norwegian CHS and well documented methods [53, 54]. Additional training would be provided as needed.

As an early intervention program, it was important to define the best recruitment point, and period of program duration in order to achieve results.

### Focus on early intervention

During the first years of the child’s life the most rapid and developmentally significant changes occur [55, 56]. At the core of health promotion and risk prevention directed at children, is the concept of early intervention [56, 57]. By supporting families through early interventions, negative outcomes can be prevented and positive development in short- and long-term promoted [20, 24, 30, 43]. The assumption is that by assessing the child’s and the family’s needs and strengths, a provision of appropriate support and service will improve the child’s and family’s outcome [57].

### The duration of the program

The PHNs at the Norwegian CHS focus on families through a universal perspective and approach. But the required tasks are time consuming and the PHNs acknowledge a need to intervene early:

*“If you are not there at the beginning, when patterns are developing, you can only try to patch and repair. Most of them learn as they go on by themselves, then there are those who are more or less occasional witnesses to their own lives, how will they master this role? If you could have a knowledge based and a follow-up, I think we could create a foundation, which people could bring with them, also when they get the next child.” (PHN).*

But it is not sufficient that this is acknowledged by the PHNs. The users of the CHS, the families, have to recognize a need for assistance.

*“I didn’t think I would become … I have always considered myself a strong person who knows how to handle things. Then the breastfeeding came along, the feeling of being weak as a mother, or something. The first time she had the growth spurt. I knew this was something to expect. But that feeling then, that I wasn’t able to feed my child; I’m a terrible mother.” (Focus group mother).*

The mothers also discussed how having their first child were accompanied with insecurity and a feeling of not knowing oneself anymore, and needing of someone to talk to, to make you feel better.

*“I didn’t know there would be all these feelings, these weird, new experiences. You want to cry, you want to talk to someone.” (Focus group mother).*

Based on the scoping review, the project group decided to focus on the first 2 years of the child’s life. Identified programs reported reduced days of infant hospitalization [23], reduced number of subsequent pregnancies, child abuse and neglect, use of welfare, and criminal behavior [29] reduction of; serious antisocial behavior, insecure attachment [24, 25] and how the home visitor and the mother formed a relationship of trust and development of increased parenting skill [33]. If the PHN could get to know the parents during pregnancy, this could help form a solid relationship and serve as a foundation for collaboration with subsequent positive short- and long -erm outcomes.

### Fit the program with the CHS system

To provide support beyond the CHS’s scheduled clinic visits [58], the PHNs described how lack of resources and the new Norwegian guidelines demanding additional services, forced them to prioritize [2, 59]. It felt morally difficult to prioritize between being adherent to the regulation of the mandatory clinical visits and being flexible and open with families requiring supplementary service. They stated that an additional program would need increased resources. It was important that the new program did not require any additional long-term courses or training for the PHNs, but could be implemented at any CHS and performed by a regular certified PHN.

### Home visit as intervention setting

The home visits provided the visitor with a better understanding of the families through the environments in which they lived, creating an understanding of the families’ need [25, 33, 36, 37, 39]. The project group discussed how the home setting might contribute to gain insight and give the PHNs a deeper understanding of the family. More important the project group needed to know if the users of the CHS accepted home visits. If the mothers did not like the idea of a PHN regularly in their home, the setting for the program had to change. Within the Norwegian CHS, one home visit within 2 weeks postpartum to all newborn is recommended [59]. Most of the focus group mothers had experienced one home visit, and compared the home visits with visits at the CHS clinic.

*“I was more open to the PHN, when talking with her at home, than going to the CHS feeling that ‘I have decided today what to do, and what to talk about.’ It is just what you want today in a way, and it is not natural communication, or a natural conversation I’d say. I don’t feel it is relaxed, as I only answer her questions. (…) But if I had a home visit, it is like the conversation is automatically more fluid.” (Focus group mother).*

The PHNs described how conducting home visits changed their perception of the mothers as they gained insight in her world.

*“You arrive at the home and the visit takes place in a small room, where the mother lives with rolled down curtains in a double bed. I’ve been at home visits like this many times. And then … there’s no living room, this is her little world. Then you understand more … what … why.” (PHN).*

One of the PHNs depicted home visits with “a picture tells more than a thousand words” analogy, allowing them to talk to the mothers on her terms, and this affected the power structure between them.

*“I’m thinking about power, the shift of power. Many of the PHNs aren’t aware of the power they have, sitting behind their desk with their door closed (..). I believe that at someone’s home, this balance shifts automatically. You get into another role. This also does something with the balance.” (PHN).*

### Continued user involvement

The project group stated that user involvement of the participating families and staff at the CHS should be an ongoing process, allowing to cross-check the program progress with reality and assure program appropriateness, both at the cultural and organizational level. This leads up to the final key element: Monitoring and evaluating the development process. Both are important tools to help ensure that the activities are implemented as planned and to assess whether desired results are achieved [60]. It was important that the process and the development of the project was a heuristic and live process, where changes would be made longitudinally based on new knowledge and project monitoring.

### The “New Mothers” program

The result was “New Mothers,” an early intervention program aimed at all first-time mothers in Stovner District. If an intervention program recruited based on risk factors only, the probability of mothers feeling stigmatized could increase [61]. “New Mothers” is based on a salutogenic theory which reduces participant stigmatization by focusing on resistance and strengths and using a universal approach. The new program will be anchored in the CHS and delivered by PHNs through home visits. Home visits as an intervention method may create a more equal power structure between a PHN and the family. The PHNs will apply MI and empathic communication as methods to captivate and connect with the mothers. First time mothers, using the CHS prenatally, will be recruited and offered home visits by a personal PHN, who follow the new expectant family with additional home visits from pregnancy until the child is 2 years. The same PHN will be responsible for providing the statutory visits at the CHS. The role of the PHNs is to support the mothers, help them identify their SOCs and use their GRRs and provide options enabling the mothers to make sound choices, benefitting the family health both in long and short-term. By establishing a trusting relationship at an early stage, the intention and hope is that the PHN can serve and support the family throughout late pregnancy and until the child turns 2 years of age.

## Discussion

Using formative research and a participatory approach an early intervention program serving a multiethnic population was successfully developed for integration in the current Norwegian CHS. The formative research cycle resembles the hermeneutic circle. Each step of the formative research cycle provided new information and gave the project group insight into relevant and different perspectives. This is in line with how the hermeneutic circle provides a deeper understanding of different parts always in reference to the whole. Development of the key elements of the program benefitted from incorporating the formative research cycle as the study design.

By using several data and information sources and user involvement at multiple levels prior to decision making, one might argue that the impact of the developers are limited. But to assume that this program was developed free of interpretation and not affected by personal values, experience or projections is not possible, and in line with the hermeneutic circle where values, experiences and projections are described as drivers to gain new knowledge [62].

The scoping review identified studies from countries lacking an existing free and low threshold CHS and all focused on targeting high risk, first time mothers. When aiming for a new program integrated in an existing universal service in a country where 98% of the eligible population attended, this would contradict the principle of universal and equal access [63]. The project group and the PHNs expressed a concern: high risk, first time mothers are not synonymous with low SOC or immigrants in need of additional services. The PHNs had experienced how apparently high-functioning first time families and mothers with few obvious risk factors faced the new and sometimes overwhelming experience of becoming parents [64, 65]. Some managed the new situation, but some needed significant additional support and advice. If the program were to only target standardized high risk, first time mothers, there would be a body of families disregarded who might have benefitted from the program [63]. The project group also feared women could feel stigmatized if immigrants or other specific groups were the only target group [66]. This could lead to a decreased recruitment; if women felt participating in the program implied they were highly likely to become a bad mother.

Early on, prior to the scoping review, the project group considered implementing a licensed early intervention program. If a program qualified for the Norwegian conditions already existed, there was no need to develop a new one. However, an existing program framework had to adapt to the universal thrust, be culturally translated and cost- effective. Some high-risk programs implemented in countries comparable to the Norwegian setting reported small intervention effects [43], and in some cases loss of support for further provision [40].

The application of a salutogenic theory provided a refocus from current paradigmatic norms of measurements of pathology and surveillance, to identification of what generates and maintains health [41, 67]. The majority of pregnant women in Norway are healthy, therefore one of the key tasks should be helping them maintain a healthy state and support their well-being. By implementing a salutogenic focus, the intention and the goal was increase the mothers SOC, self-efficacy and motivation to positively make necessary changes affecting the family’s’ life course and health [41, 44].

Relationship and trust are core elements in early intervention programs [30, 34], and a core concept of the Norwegian CHS [68]. The majority if the interventions detected in the scoping review recruited the mothers prenatally and potentially build a relationship at an early stage. In a study by Zapart [36], the participants reported a positive relationship with the home visitor characterized by availability and responsiveness as the key to a long-term impact of the program. This can be explained by the concept of “parallel process” [69].

In Norway the PHC physicians and midwives are currently the only professionals from the CHS who have contact with the pregnant women until birth. Their responsibilities were not altered because of the New Mothers program, but the PHNs had concerns about how the mothers would interpret meeting the PHN during pregnancy. If they were to meet the mothers prenatally, it was important for PHNs to be clear about the intention of the home visits, and not overstep their professional responsibility related to routine medical and prenatal care.

Another important premise was to use the home as the delivery setting. A wide range of health and social problems can be addressed by nurses visiting mothers pre- and postnatally at home [32, 40, 70], promoting an environment conductive for maternal and child health, and long-term physical and psychological well-being [30]. If the environment positively changes the power structure between the professional and the client, it enables the mothers to be more in control. The PHNs described how being a visitor made them feel humble without losing their professional role. One of the PHN described this as: “personal, but never private.” Home visits strengthens the connection, and facilitates learning and support [33].

A new and revised guideline for the Norwegian CHS, from 2017, supports an increase in home visits as setting for contact, trust and observation [59]:

*“The CHS is to consider home visits as a supplementary offer directed at families with additional needs. The home visit can improve contact and trust, additional to observation of skills and interaction between child, siblings and parents in their environment.” p.90.*

To meet the escalating demand for service and the rapid demographical changes it is necessary to meet the users of the CHS at their terms, define their needs, strengths and help them individually. This means tailoring the service, and for the PHNs to work close and in collaboration with the mothers. The statutory visits at the CHS will continue, but for first time mothers the connection to their PHN will start prenatal and they will receive a closer follow up.

Throughout this development period the project group has learned the necessity of user participation and systematic collection of information. The group has also built and tailored a new program to the Norwegian context.

### Limitations and strengths

The focus groups with the mothers and the PHNs provided insight and strengthened the user involvement of the program development. A limit is that the members might not have expressed their honest thoughts, especially if their thoughts opposed the views of another member. However, focus group can generate more in depth information and rich narratives. We did not experience any language challenges, although not all mothers spoke fluent Norwegian; language differences might have limited the level of participation and engagement. The moderator tried to avoid impacting the outcome of the interviews, by focusing on that the intention of user involvement was to give a voice to the users in order to develop a program that better fitted the needs of the families and mothers in the district.

## Conclusion

To the best of our knowledge we did not identify any other published studies describing the development of an early intervention program aimed at maternal and child health employing user participation. This process seems to go unaddressed within the literature. Decision making, program theory and why programs use certain key elements and strategies, are rarely described, and we hereby provide our contribution to this knowledge gap.

Formative research as study design provided information and gave directions on how to frame a CHS program. It allowed us to explore data collected from different sources, identified facilitators and barriers of key program elements, utilized user participation, and identified power structures. Interviews and focus groups provided insight on challenges, reflections and contributed with germane information for the proposed framework and secured user involvement. The scoping review provided directions on key program elements, experiences and research results. Monthly meetings with the project group enabled feedback loops’ on data and information collected that secured immersion and continued the framework development.

It is, however, essential to stress the importance that this program is still developing. The next step of the process is piloting the program in Stovner. User participation will keep evolving in this phase and changes will be made to ensure culturally adaptation and user involvement, and all will be documented. There is a need to both evaluate the program qualitatively and quantitatively. The “New Mothers” program will be evaluated qualitatively, focusing at parents’ self-efficacy and program satisfaction, but also whether the program or not added a new, and hopefully positive, dimension to the CHS and the PHNs professional role.

## Abbreviations

*CHS*: Child Health Service
*CWS*: Child Welfare Services
*PHC*: Primary Health Care service
*PHN*: Public Health Nurse
